# Elective Heparin-Free Veno-Venous Extracorporeal Membrane Oxygenation for High-Risk Airway Management in Advanced Laryngeal Tumor Obstruction: A Case Report

**DOI:** 10.3390/jcm15114365

**Published:** 2026-06-04

**Authors:** Joanna Prokop, Konrad Zuzda, Wojciech Jan Górski, Miłosz Jankowski, Eliza Brożek-Mądry, Konstanty Szułdrzyński

**Affiliations:** 1 Department of Anaesthesiology and Intensive Care, National Medical Institute of the Ministry of the Interior and Administration, 02-507 Warsaw, Poland; joanna.prokop@pimmswia.gov.pl (J.P.); wojciech.gorski@pimmswia.gov.pl (W.J.G.); milosz.jankowski@pimmswia.gov.pl (M.J.); konstanty.szuldrzynski@pimmswia.gov.pl (K.S.); 2 Department of Otorhinolaryngology, National Medical Institute of the Ministry of the Interior and Administration, 02-507 Warsaw, Poland; eliza.brozek@pimmswia.gov.pl

**Keywords:** VV-ECMO, tracheostomy, laryngeal carcinoma

## Abstract

**Background:** Critical upper airway obstruction caused by advanced laryngeal malignancy poses an extreme risk of airway loss during induction of anesthesia and instrumental airway management. Veno-venous extracorporeal membrane oxygenation (VV-ECMO) has emerged as a strategy to enable safe performance of high-risk airway interventions. However, reports on heparin-free protocols in this setting remain scarce. **Methods:** A 46-year-old male with progressive dyspnea caused by a large laryngeal tumor reducing the residual glottic lumen to ~1 mm was admitted urgently. Safe endotracheal intubation was deemed impossible. Elective heparin-free VV-ECMO was established under local anesthesia via percutaneous femoro-femoral cannulation before induction of general anesthesia. **Results:** Under ECMO support, a technically demanding tracheostomy and tumor biopsy were performed without hypoxemic episodes. VV-ECMO was maintained postoperatively for 48 h without systemic anticoagulation and was weaned without hemorrhagic or thrombotic complications. Histopathology confirmed squamous cell carcinoma grade 2; the patient was discharged home after initiation of systemic immunotherapy. **Conclusions:** Elective heparin-free VV-ECMO can provide effective and safe respiratory support for patients with critical airway obstruction undergoing high-risk airway procedures. Pre-emptive cannulation under local anesthesia, femoro-femoral access in anatomically compromised necks, and short heparin-free circuit runs mitigate both airway and hemorrhagic risk. Prospective studies are needed to establish standardized patient selection criteria and anticoagulation protocols.

## 1. Introduction

Critical upper airway obstruction represents one of the most dangerous emergencies in anesthesia and critical care, rapidly progressing to severe hypoxemia, hypercapnia, and cardiovascular collapse. Conventional airway management strategies, including endotracheal intubation, supraglottic devices, and jet ventilation, may fail or carry an unacceptable risk of a “cannot ventilate, cannot oxygenate” scenario [1].

Patients with laryngeal or hypopharyngeal tumors frequently present with glottic narrowing, distorted anatomy, and intolerance to the supine position, making airway instrumentation hazardous [2]. In such cases, extracorporeal oxygenation can provide respiratory support independent of airway patency, creating controlled conditions for tracheostomy, bronchoscopy, or airway stenting [3].

Veno-venous ECMO (VV-ECMO) has become the preferred modality for isolated respiratory support during high-risk airway interventions, and its pre-emptive use before airway manipulation appears safer than rescue ECMO after respiratory collapse [3,4]. The 2022 American Society of Anesthesiologists (ASA) Practice Guidelines for Management of the Difficult Airway recognize ECMO as a valid option, recommending its initiation “when/if appropriate and available” [1].

Anticoagulation constitutes a critical concern in this setting: standard heparin-based ECMO protocols substantially increase hemorrhagic risk during upper airway surgery. Short-term heparin-free VV-ECMO protocols have been reported in patients with contraindications to anticoagulation [4,5]; however, evidence remains limited. Here we report the successful application of elective heparin-free VV-ECMO as a bridge to definitive airway management in a patient with critical obstruction caused by advanced laryngeal malignancy.

## 2. Case Presentation

This case was managed at a tertiary referral center with a dedicated multidisciplinary airway team with around-the-clock ECMO capability. A 46-year-old male with long-term nicotine dependence, alcohol use disorder, liver cirrhosis, and polyneuropathy was admitted urgently to the Department of Otolaryngology with progressive dyspnea at rest. Over the preceding two months he had developed a painful, enlarging neck mass on the left side with worsening respiratory symptoms. Dyspnea was markedly exacerbated in the supine position, raising concern for impending complete airway obstruction.

Flexible fiberoptic laryngoscopy revealed a large exophytic tumor originating from the left pyriform sinus, completely occupying the glottic inlet. Direct visualization of the vocal cords and glottic aperture was impossible. Computed tomography (CT) of the neck (Figure 1) demonstrated a large tumor mass (52 × 28 × 100 mm) extending from the mandibular angle to above the sternoclavicular joint, causing marked narrowing of the oropharyngeal and laryngeal airway with rightward displacement of the larynx and trachea, and infiltration of the left thyroid lobe, hyoid bone, and laryngeal cartilages. A suspicious lesion (39 × 18 × 17 mm) in the right submandibular region raised concern for regional metastasis. The residual airway diameter at the glottic level was approximately 1 mm. The patient was deemed at extremely high risk of complete airway obstruction during induction of general anesthesia.

Following multidisciplinary discussion among anesthesiologists, otolaryngologists, and intensivists, alternative airway management strategies were systematically evaluated. Awake fiberoptic intubation was excluded due to the critical airway narrowing to approximately 1 mm, which precluded safe passage of any endotracheal tube [1]. Awake tracheostomy without extracorporeal support was considered prohibitively high-risk given the extensive tumor mass infiltrating subcutaneous tissues, neck musculature, and thyroid gland; any significant hemorrhage during dissection could have precipitated complete airway obstruction and respiratory arrest in a patient with no reserve [6]. Jet ventilation was not feasible as percutaneous airway access through the tumor-infiltrated neck was anatomically impossible [3]. Standby ECMO was deemed inappropriate due to the time to set up the device and establish cannulation. In the event of complete airway collapse during induction, the delay would have resulted in unacceptable duration of hypoxia. Similarly, rescue ECMO after respiratory collapse was considered too hazardous given the critical baseline status.

Therefore, a decision was made to perform urgent tracheostomy under elective pre-emptive VV-ECMO support, initiated before induction of general anesthesia. This approach has been recommended for patients with near-occlusion of the airway, as it allows for safe completion of airway intervention under controlled conditions. Due to the anticipated high bleeding risk from tumor infiltration and extensive surgical dissection, a heparin-free ECMO strategy was planned from the outset [5].

VV-ECMO cannulation was performed in the operating room under local anesthesia with continuous ultrasound guidance. The ECMO system comprised a Maquet Cardiohelp console with an HLS 7.0 Advanced oxygenator featuring BioLine antithrombotic coating (Getinge, Gothenburg, Sweden), selected for its suitability for short-term procedural support. Percutaneous femoro-femoral cannulation was achieved without complications (Figure 2). A 25 Fr drainage cannula was introduced via the left femoral vein with its tip positioned in the inferior vena cava (IVC), and a 23 Fr return cannula was placed via the right femoral vein with its tip in the right atrium (RA). Cannula positions were confirmed ultrasonographically, with adequate tip separation of ~9 cm verified to minimize recirculation risk, and general anesthesia was subsequently induced.

Following initiation of VV-ECMO, a staged approach to extracorporeal support was employed. Initially, while the patient remained awake and breathing spontaneously, ECMO was maintained at minimal settings: blood flow of 2.0 L/min, sweep gas flow of 0 L/min, and FiO_2_ of 0.3. This configuration provided circulatory standby without active gas exchange, preserving the patient’s native respiratory function while ensuring immediate availability of full extracorporeal support. Upon induction of general anesthesia and cessation of spontaneous ventilation, ECMO settings were escalated to meet the patient’s full gas exchange requirements. Inspired oxygen fraction and sweep gas flow in the ECMO oxygenator were tailored to achieve proper gas exchange with physiologic values of PaO_2_ and PaCO_2_ in arterial blood gas analysis. Recirculation was continuously assessed by comparing circuit venous oxygen saturation (SvO_2_) with peripheral oxygen saturation, with no clinically significant recirculation detected throughout the procedure.

Given the heparin-free approach central to this case, baseline coagulation parameters were documented prior to cannulation: aPTT 29.3 s, PT 10.6 s, INR 0.96, and platelet count 197 × 10^9^/L. No systemic anticoagulation was administered throughout the ECMO run. Regular circuit inspections revealed no visible thrombus formation, and oxygenator performance remained stable without efficiency loss. The patient required no blood product transfusion before, during, or after the procedure.

Tracheostomy proved technically challenging due to extensive tumor infiltration of the subcutaneous tissue, neck musculature, and thyroid gland, combined with tracheal displacement and rotation to the right. Tissues were fragile, bleeding-prone, and adherent. After careful dissection, the trachea was incised and a size 8 tracheostomy tube was successfully inserted. Hemostasis was achieved, and tumor biopsy specimens were obtained. Throughout the procedure, VV-ECMO provided stable gas exchange without hypoxemic episodes.

Postoperatively, the patient was transferred to the intensive care unit (ICU) and initially received mechanical ventilation via the tracheostomy tube. Sedation was gradually tapered; ventilation was weaned from assist-control mode to continuous positive airway pressure (CPAP) with pressure support and subsequently discontinued as spontaneous breathing recovered. VV-ECMO was maintained at a blood flow of 2.0 L/min without systemic anticoagulation. The patient remained hemodynamically stable without vasopressor support, and no bleeding complications at surgical or cannulation sites were observed.

On the second day in the ICU, VV-ECMO was successfully discontinued, and cannulas were removed without complications. The patient remained stable on spontaneous ventilation via tracheostomy with supplemental oxygen and was transferred the following day to the Department of Otolaryngology for continued oncological evaluation.

Histopathological examination confirmed squamous cell carcinoma grade 2 of the hypopharynx, originating from the left pyriform sinus. This finding was consistent with the initial fiberscope assessment revealing an exophytic tumor arising from this location. Squamous cell carcinoma represents the most common primary malignancy requiring ECMO-supported airway interventions, accounting for a significant proportion of cases in published series [4].

Following multidisciplinary tumor board discussion, the patient was disqualified from surgical resection and radiotherapy. Systemic immunotherapy with nivolumab (anti-PD-1 immune checkpoint inhibitor) was initiated. Percutaneous endoscopic gastrostomy (PEG) was subsequently performed to ensure adequate nutritional support. The patient was discharged home in stable condition.

## 3. Discussion

This case demonstrates the successful application of elective heparin-free VV-ECMO as a bridge to definitive airway management in a patient with critical upper airway obstruction caused by advanced laryngeal malignancy, an approach consistent with current recommendations emphasizing early identification of high-risk patients and multidisciplinary planning in experienced centers [1,3].

Extracorporeal oxygenation has become a valuable tool in managing complex airway pathology. Studies have reported successful ECMO use during open tracheal resections and extended tracheobronchial reconstructions, with VV-ECMO increasingly preferred for isolated respiratory support [3,8]. Stokes et al. reported a series of VV-ECMO-supported high-risk airway interventions with high procedural success rates and acceptable complication profiles [9]. Maxwell and Forrest provided a comprehensive review of ECMO indications and management protocols in airway procedures [6]. The ASA 2022 guidelines formally endorsed ECMO as an option in the cannot-intubate-cannot-oxygenate scenario [1], reflecting growing evidence for its role as a safety mechanism during high-risk airway interventions [9].

A critical aspect of our approach was the omission of systemic anticoagulation. Standard ECMO circuits require heparin to prevent thrombosis; however, this substantially increases hemorrhagic risk during vascular and airway surgery [5]. Sun et al. reviewed heparin-free VV-ECMO specifically for airway obstruction and found that short-duration circuits could be run safely without thrombotic complications, with literature indicating that the median safe time for ECMO without systemic anticoagulation ranges from 70 to 114 h [5]. Importantly, our patient did not experience other ECMO-associated adverse events such as infection, neurological injury, cardiac complications, or low-perfusion organ damage.

Regarding patient demographics in the existing literature, published case series have included patients with varying comorbidity profiles and age ranges. In the largest single-center study by Kim and colleagues, patients undergoing ECMO-supported airway interventions ranged from young adults to elderly individuals with malignant conditions [3,6]. Hong et al. reported on 18 patients with central airway obstruction caused by malignant disease, with a 60-day mortality of 27.8%, reflecting the severity of underlying pathology rather than ECMO-related complications [10]. Stokes et al. described a cohort with median age of 59 years (range 33.8–68.0), including patients with significant comorbidities undergoing high-risk bronchoscopy [9]. Our patient, despite being relatively young at 46 years, presented with substantial comorbidities including liver cirrhosis and polyneuropathy, which influenced our conservative postoperative ECMO management strategy. Nevertheless, the 48 h heparin-free course was uneventful, with no hemorrhagic or circuit-related complications, consistent with previously published cases.

The feasibility of pre-emptive VV-ECMO cannulation under local anesthesia represented another important element. Femoro-femoral access was particularly suitable in our patient, whose neck was diffusely infiltrated by tumor, making jugular cannulation anatomically hazardous. While femoro-jugular configuration is generally preferred as it can provide higher flow rates [6], femoro-femoral access offers distinct advantages in patients with orthopnea, as it can safely be established in the reverse Trendelenburg position [6]. The primary limitation of bifemoral cannulation is the increased risk of extracorporeal recirculation of already oxygenated blood, which inevitably limits the amount of oxygen delivered to the patient [4]. To mitigate this risk, adequate tip separation of at least 7 cm between drainage and return cannula is essential [6]. In our case, tip separation of ~9 cm was confirmed ultrasonographically, and blood flow provided adequate gas exchange throughout the procedure. This strategy avoids the risks associated with general anesthesia induction prior to securing oxygenation [9] and represents a key safety step we recommend in similar high-risk presentations where neck anatomy is compromised.

We decided on to maintain VV-ECMO into the postoperative period rather than performing immediate decannulation as practiced in high-volume centers. This reflected our center’s limited experience with this specific indication, combined with the patient’s underlying hepatic dysfunction and hemodynamic complexity. Although this prolonged overall ECMO exposure, no thrombotic complications were encountered.

This report has several limitations inherent to a single case description. Broader conclusions regarding heparin-free VV-ECMO for airway obstruction require prospective multicenter data. The CAse REport (CARE) guidelines were followed in the preparation of this report [11].

## 4. Conclusions

In conclusion, this case supports the feasibility of elective heparin-free VV-ECMO as respiratory support in a carefully selected patient with critical airway obstruction undergoing high-risk airway intervention. The key elements of this approach included: early multidisciplinary identification of prohibitive airway risk; pre-emptive ECMO cannulation under local anesthesia before induction; femoro-femoral access when the neck was anatomically compromised; and a short heparin-free circuit run to mitigate surgical hemorrhagic risk. However, generalization of these findings is limited by the single-case nature of this report. Prospective multicenter studies are needed to establish standardized patient selection criteria and anticoagulation protocols for this indication.

## Figures and Tables

**Figure 1:**
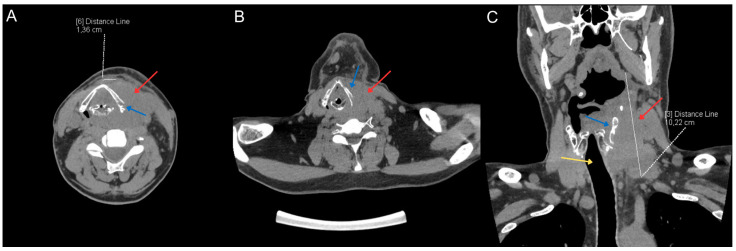
Non-contrast computed tomography of the neck ((**A**,**B**), sagittal plane; (**C**), coronal plane) demonstrating a large tumor mass 52 × 28 × 100 mm (red arrow) causing critical narrowing of the laryngopharynx with rightward displacement of the larynx and trachea (yellow arrow). The mass infiltrates of the left greater horn of hyoid bone, thyroid lobe, and laryngeal cartilages (blue arrow).

**Figure 2:**
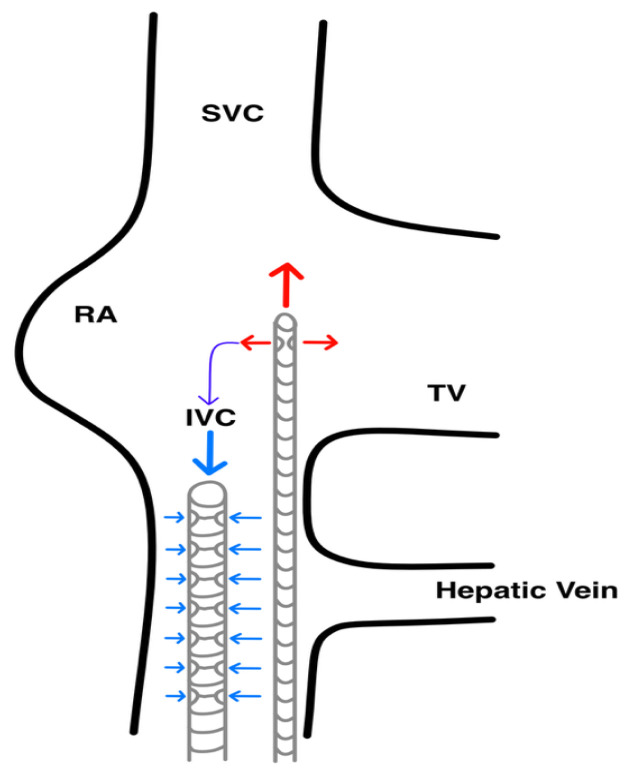
VV-ECMO cannula positioning. Drainage cannula: left femoral vein, tip in the IVC. Return cannula: right femoral vein, tip in the RA. Tip separation of ~9 cm prevented recirculation [7]. IVC—inferior vena cava; RA—right atrium; TV—tricuspid valve.

## Data Availability

All data generated or analyzed during this study are included in this published article. No datasets were deposited in public repositories.

## Abbreviations

The following abbreviations are used in this manuscript:

aPTT	Activated Partial Thromboplastin Time
ASA	American Society of Anesthesiologists
CARE	CAse REport (guideline standard)
CPAP	Continuous Positive Airway Pressure
CT	Computed Tomography
ECMO	Extracorporeal Membrane Oxygenation
FiO_2_	Fraction of Inspired Oxygen
Fr	French (catheter size)
ICU	Intensive Care Unit
INR	International Normalized Ratio
IVC	Inferior Vena Cava
PaCO_2_	Partial Pressure of Arterial Carbon Dioxide
PaO_2_	Partial Pressure of Arterial Oxygen
PD-1	Programmed Death-1 (immune checkpoint receptor)
PEG	Percutaneous Endoscopic Gastrostomy
PT	Prothrombin Time
RA	Right Atrium
SvO_2_	Venous Oxygen Saturation (circuit)
TV	Tricuspid Valve

## References

[1] Apfelbaum J.L., Hagberg C.A., Connis R.T., Abdelmalak B.B., Agarkar M., Dutton R.P., Fiadjoe J.E., Greif R., Klock P.A., Mercier D. (2022). 2022 American Society of Anesthesiologists Practice Guidelines for Management of the Difficult Airway. Anesthesiology.

[2] Abdelmalak B.B. (2023). The Role of Extracorporeal Membrane Oxygenation in the Management of the Difficult Airway. J. Cardiothorac. Vasc. Anesth..

[3] Pořízka M., Michálek P., Votruba J., Abdelmalak B.B. (2021). Extracorporeal Oxygenation Techniques in Adult Critical Airway Obstruction: A Review. Prague Med. Rep..

[4] Slama A., Stork T., Collaud S., Aigner C. (2023). Current Use of Extracorporeal Life Support in Airway Surgery: A Narrative Review. J. Thorac. Dis..

[5] Sun B., Zhou M., Wang R., Liu Q., Yan L., Zhang Y., Zhang J., Wang L. (2024). Heparin-Free Veno-Venous ECMO for Airway Obstruction: A Case Report and Review of Literature. Medicine.

[6] Maxwell C., Forrest P. (2023). The Role of ECMO Support in Airway Procedures. BJA Educ..

[7] Burrell A.J.C., Ihle J., Pellegrino V.A., Sheldrake J., Nixon P.T. (2018). Cannulation Technique: Femoro-Femoral. J. Thorac. Dis..

[8] Hoetzenecker K., Klepetko W., Keshavjee S., Cypel M. (2017). Extracorporeal Support in Airway Surgery. J. Thorac. Dis..

[9] Stokes J.W., Katsis J.M., Gannon W.D., Rice T.W., Lentz R.J., Rickman O.B., Avasarala S.K., Benson C., Bacchetta M., Maldonado F. (2021). Venovenous Extracorporeal Membrane Oxygenation during High-Risk Airway Interventions. Interact. Cardiovasc. Thorac. Surg..

[10] Hong Y., Jo K.-W., Lyu J., Huh J.W., Hong S.B., Jung S.-H., Kim J.H., Choi C.M. (2013). Use of Venovenous Extracorporeal Membrane Oxygenation in Central Airway Obstruction to Facilitate Interventions Leading to Definitive Airway Security. J. Crit. Care.

[11] Gagnier J.J., Kienle G., Altman D.G., Moher D., Sox H., Riley D. (2014). The CARE Guidelines: Consensus-Based Clinical Case Report Guideline Development. J. Clin. Epidemiol..

